# Function and regulation of tau conformations in the development and treatment of traumatic brain injury and neurodegeneration

**DOI:** 10.1186/s13578-016-0124-4

**Published:** 2016-12-05

**Authors:** Onder Albayram, Megan K. Herbert, Asami Kondo, Cheng-Yu Tsai, Sean Baxley, Xiaolan Lian, Madison Hansen, Xiao Zhen Zhou, Kun Ping Lu

**Affiliations:** Division of Translational Therapeutics, Department of Medicine and the Cancer Center, Beth Israel Deaconess Medical Center, Harvard Medical School, 330 Brookline Avenue, CLS 0408, Boston, MA USA

## Abstract

One of the two common hallmark lesions of Alzheimer’s disease (AD) brains is neurofibrillary tangles (NFTs), which are composed of hyperphosphorylated tau protein (p-tau). NFTs are also a defining feature of other neurodegenerative disorders and have recently been identified in the brains of patients suffering from chronic traumatic encephalopathy (CTE). However, NFTs are not normally observed in traumatic brain injury (TBI) until months or years after injury. This raises the question of whether NFTs are a cause or a consequence of long-term neurodegeneration following TBI. Two conformations of phosphorylated tau, *cis* p-tau and *trans* p-tau, which are regulated by the peptidyl-prolyl isomerase Pin1, have been previously identified. By generating a polyclonal and monoclonal antibody (Ab) pair capable of distinguishing between *cis* and *trans* isoforms of p-tau (*cis* p-tau and *trans* p-tau, respectively), *cis* p-tau was identified as a precursor of tau pathology and an early driver of neurodegeneration in AD, TBI and CTE. Histological studies shows the appearance of robust *cis* p-tau in the early stages of human mild cognitive impairment (MCI), AD and CTE brains, as well as after sport- and military-related TBI. Notably, *cis* p-tau appears within hours after closed head injury and long before other known pathogenic p-tau conformations including oligomers, pre-fibrillary tangles and NFTs. Importantly, *cis* p-tau monoclonal antibody treatment not only eliminates *cis* p-tau induction and tau pathology, but also restores many neuropathological and functional outcome in TBI mouse models. Thus, *cis* p-tau is an early driver of tau pathology in TBI and CTE and detection of *cis* p-tau in human bodily fluids could potentially provide new diagnostic and prognostic tools. Furthermore, humanization of the *cis* p-tau antibody could ultimately be developed as a new treatment for AD, TBI and CTE.

## Background

Tau protein is a member of the microtubule-associated family of proteins which are expressed predominantly in the brain. Tau’s primary functions, which include the stabilization of microtubules and the coordinated movement of molecules along the microtubule, are tightly regulated by phosphorylation [1–3]. In its native state, tau is present in a stable, unfolded monomeric conformation. However, via as yet unknown mechanisms, tau becomes aberrantly phosphorylated, or hyperphosphorylated, and aggregated in several neurodegenerative diseases, collectively known as tauopathies [2, 3].

Although human tau is predominantly expressed in neurons, it can also be found in astrocytes and oligodendrocytes [4]. In the central nervous system, alternate splicing of exon 2, 3 and 10 leads to the generation of six isoforms of tau which range from 352 to 441 amino acids in length and 60–74 kDa in weight as determined by SDS-PAGE analyses [5]. The variability in these isoforms derives from the presence or absence of exon inserts (0, 1 or 2) in tau’s N-terminal region and the presence or absence of microtubule binding repeat domains in tau’s C-terminal region [6]. Highly phosphorylated tau with a 3-repeat domain in the C-terminus predominates during early stages of development whereas a ~ 1:1 ratio of 3-repeat:4-repeat tau is present in adults. Trace amounts of tau are also detectable in peripheral organs such as the heart, kidney, lungs, muscle, pancreas and testis but this peripheral tau is larger than brain tau with an additional N-terminal sequence tau encoded by exon 4A. Thus, it is sometimes referred to as ‘big tau’ [6].

Phosphorylation of tau decreases normally with age and coincides with the development of phosphatases [7]. However, in tauopathies, the aberrant phosphorylation of tau leads to abnormal accumulations of tau in the brain [8]. The most archetypal tau aggregations occur in Alzheimer’s disease (AD) in which hyperphosphorylated tau forms aggregates within the cell bodies known as neurofibrillary tangles (NFTs). In addition to AD there are several well-established neurodegenerative tauopathies, which include fronto-temporal dementia, Pick’s disease, amyotrophic lateral sclerosis (ALS), progressive supranuclear palsy (PSP) and corticobasal dementia (CBD) as well as Parkinson’s disease with dementia [9]. More recently, abnormal accumulations of tau have been associated with chronic traumatic encephalopathy (CTE) and traumatic brain injury (TBI), particularly in sports-related injuries exposing athletes to repeated mild traumatic brain injury (rmTBI), with or without concussion, and military personnel exposed to repeated explosive blast injuries [10–12].

## Post-translational modifications and tau aggregation in neurodegenerative diseases

Tau can be post-translationally modified in several ways including phosphorylation, acetylation, glycation, prolyl-isomerization, cleavage or truncation, nitration, polyamination, ubiquitination, sumoylation, oxidation and aggregation [13–15]. The most well studied of these, and arguably one of the most important, is the phosphorylation of tau [16]. Tau in its dephosphorylated state is not prone to aggregation but phosphorylation of tau, which is required for regulation of its physiological functions, can increase or reduce its stability. Although debate continues regarding the contribution of tau to neurodegeneration, there is mounting evidence to suggest that the phosphorylation of tau, or more notably the hyper-phosphorylation of tau, may lead to its increased aggregability, eventually leading to neuropathogenicity such as NFTs [17, 18]. In AD, hyperphosphorylated tau in NFTs is composed of approximately equal amounts of both 3R and 4R tau whereas other diseases are associated with a higher ratio of 3R:4R (Pick’s disease) or a higher ratio of 4R:3R (CBD and PSP). The appearance and localisation of tau aggregates in different neurodegenerative diseases also varies widely [19, 20].

## Hyperphosphorylation of tau

The primary mode of regulation of tau function occurs via phosphorylation at specific sites and coordinated phosphorylation events are necessary for proper neurite outgrowth and axonal transport processes [21]. While the exact role of abnormal phosphorylation of tau in tauopathies is unclear, there is substantial evidence to show that hyperphosphorylation plays a key role in the development of AD and a myriad of other neurodegenerative diseases associated with pathological tau [18, 22]. Hyperphosphorylation induces the formation of tau aggregates with highly phosphorylated tau showing self-assembly in vitro. Mutations at the Ser422 location lead to a significant increase in tau-tau propensity [17]. Pre-tangle formation in specific brain regions has been shown to hinder memory, disrupt synaptic function, and produce a rapid impairment of long-term potentiation (LTP). These toxic molecules can also catalyse neuronal loss and the accumulation of intracellular neurofibrillary tangles (NFTs) composed mainly of tau protein [17, 23]. Growing evidence also proposes that these potentially neurotoxic conglomerations perhaps act in a prion-like manner, but additional research will be required to elucidate their exact mechanism of action [24]. Interestingly, hyperphosphorylated tau aggregates reach a neurotoxic state prior to the formation of neurofibrillary tangles, thus making tau an enticing new target for preventative treatments of tau pathology [25]. Along with increased tau-tau affinity, hyperphosphorylation has also been correlated with a loss of normal tau physiological function. Neurons labeled with antibodies to recognize phospho-Thr231 and phospho-Ser262 [26] display significantly decreased normal physiological interactions of tau with microtubules upon phosphorylation [25, 27] greatly diminishing the ability of this protein to bind to, as well as stabilize, microtubules. It is therefore suggested that the loss of normal physiological functions, as well as the onset of toxic characteristics driven by the aberrant phosphorylation of tau, likely contribute synergistically to tau pathology.

## Tau in traumatic brain injury and chronic traumatic encephalopathy

TBI has been identified as a major risk factor for CTE and AD, which are characterized by abnormally phosphorylated tau aggregates in neurons and glia of multiple brain regions [28, 29]. For example, one in three NFL players are expected to experience cognitive problems in their lifetime. TBI is a leading cause of death or disability of children and young adults aged 1–44 and also affects one in five veterans of the Afghanistan and Iraq conflicts [29]. However, pathogenic mechanisms leading from acute TBI to chronic neurodegeneration are virtually unknown. There is no effective treatment available for mitigating secondary injury after acute TBI and preventing the late development of AD or CTE. Neurofibrillary tangles (NFTs) composed of phosphorylated tau are a neuropathological hallmark not only of AD but also CTE [11, 29]. Furthermore, the tau isoform and hyperphosphorylation profiles of tau tangles purified from boxer CTE brains and AD brains are indistinguishable [30, 31]. Tau in these diseases is commonly hyperphosphorylated on Ser or Thr residues, especially those preceding a Pro residue (pSer/Thr-Pro) and such phosphorylation precedes tangle formation and neurodegeneration. However, it is not fully understood how phosphorylation causes tau, which serves a vital physiologic function in healthy neurons, to become pathogenic. Until recently, whether tau is further regulated after phosphorylation and if the pathogenic phosphorylated tau can be blocked without affecting physiological tau were not known.

In rodent models mimicking the effects of TBI, a notable increase in total tau levels and p-tau pretangle conformations, in conjunction with white matter degradation and increased neuroinflammation, have been found 2–3 months after post-injury [32]. These findings indicate a potential role for tau in the propagation of neuropathology in TBI and illustrate a possible bridge between TBI and CTE.

## The emergence of a new tau isoform—*cis* p-tau

Proline-directed Ser/Thr phosphorylation is a central common signaling mechanism in the cell. We have found that certain pSer/Thr-Pro motifs exist in *cis* or *trans* conformations, and their conversion and function is further regulated by the unique prolyl isomerase Pin1 [33]. It has been shown that Pin1 is pivotal in protecting against age-dependent tau aggregation in AD by isomerizing the phosphorylated T231-P motif in tau (pT231-tau or p-tau) from *cis* to *trans* p-tau [33–35]. Furthermore, Pin1 is required for the regulated dephosphorylation of tau during microtubule stabilisation. Therefore, in the absence of Pin1, tau cannot be properly dephosphorylated and the ratio of *cis* p-tau *to trans* p-tau ratio is increased [33]. This discovery indicates that *cis* p-tau may be pathologically important and targetable. Normally, more than 90% of conventional pT231-tau peptides are in *trans* p-tau conformation [36] and therefore can only be used to generate trans mAbs [36, 37], which would likely reduce physiological tau. Using novel peptide chemistry, we recently generated not only *trans* p-tau-specific but also *cis* p-tau-specific antibodies which we used to discover a previously unrecognized pathogenic *cis* p-tau which appears early-on in the disease process and which is not readily or dynamically converted to *trans* in vivo in the absence of Pin1 [37].

Deficits in the function of Pin1, correlating with an increase in *cis*- p-tau have already been implicated in early AD [37, 38]. In particular, Pin1 down-regulation [33, 35], Cys113 oxidation [39] and phosphorylation at residue S71 [39, 40] have been shown to contribute to the aberrant tau function in AD. Down-regulation of Pin1 via serum depletion or Cys113 oxidation via hypoxia in cultured neurons correlates well with increases in *cis* p-tau. Pin1 binds to tau primarily at the T231 residue catalysing the conversion from *cis* to *trans* p-tau [33, 41]. Since the phosphorylation of T231 is also an early step in tangle formation [42], Pin1 plays a critical role in reversing phosphorylation, preventing hyperphosphorylation and preventing accumulation of *cis* p-tau [33, 37, 38]. We further discovered that *cis*, but not *trans*, p-tau appears early in the neurons of human brains affected by MCI. We found that *cis* p-tau accumulates exclusively in the dystrophic neurites of degenerating neurons as AD progresses and correlates well with cognitive deficits [38]. Moreover, *cis*, but not *trans*, p-tau loses its normal microtubule-assembling ability, and gains toxic function, being resistant to dephosphorylation and degradation, and prone to aggregation [37, 38, 43]. Thus, *cis* p-tau may be a precursor of tau pathologies and an early driver of neurodegeneration which may have the potential for neuron-to-neuron transmission in TBI, CTE and AD.

**Fig. 1:**
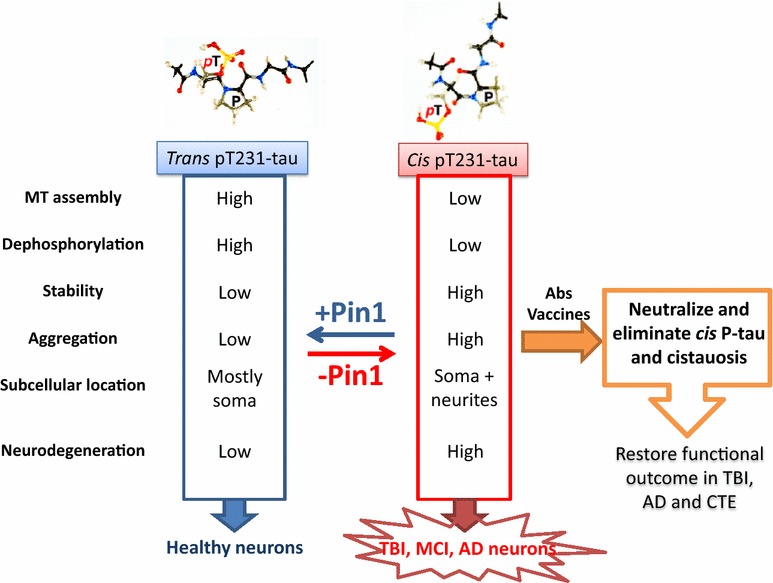
Pin1 prevents the accumulation of the pathogenic *cis* P-Tau by converting it to non-pathogenic *trans* form. pT231-tau exists in the two-distinct *cis* and *trans* conformations, as depicted in cartoons of the primary backbone structures. *Cis*, but not *trans*, pT231-tau loses normal function and gains pathogenic function. Pin1 prevents the accumulation of the pathogenic *cis* pT231-tau conformation by converting it into the nonpathogenic *trans* form. Conformation specific antibodies against the pathogenic *cis* pT231-tau might be developed for the treatment of AD, MCI, TBI, CTE or other kind of tauopathies, during its early stages. *AD* Alzheimer’s disease; *MCI* mild cognitive impairment; *TBI* traumatic brain injury; *CTE* chronic traumatic encephalopathy

Using *cis* and *trans* tau-specific monoclonal antibodies it has been possible to show that robust *cis* p-tau appears early in human MCI, AD and CTE brains, as well as after sport- and military-related TBI and long before tau aggregates or fibrils can be detected [43]. In TBI closed-head injury mouse models, *cis*, but not *trans*, p-tau was readily induced at 12 h post-injury and further increased with time, localizing mainly to axons leading to axonopathy. This occurred long before commonly other known pathological tau, such as fibrillary tau and tau oligomers, could be detected. Furthermore, *cis* p-tau monoclonal antibody (mAb) treatment eliminated *cis* p-tau induction as well as *cis* p-tau-induced neurotoxicity in stressed neuron models in vitro and in TBI mouse models in vivo [43]. Of note, recent studies have shown the presence of tau oligomers within 48 h of severe open-head injury in mouse models [44], which were not observed in our closed-head injury model [43] (and unpublished results). Thus, neutralizing mAbs against the early and toxic *cis* p-tau reduce pathological tau without affecting healthy *trans* p-tau, and have the potential to be highly efficacious and specific in halting or preventing tau pathology and memory loss in TBI, CTE and AD at early stages (Fig. 1). The mechanism by which TBI induces robust *cis* p-tau induction is currently unclear. However, it is likely that TBI, as in stroke and AD, causes the induction of the stress protein death-activated protein kinase 1 (DAPK1), which is responsible for the phosphorylation and inactivation of Pin1 [40]. Therefore, increases in DAPK1 will likely cause inactivation of Pin1 and subsequent induction of *cis* p-tau.

## Discovery of cistauosis and its role in early common disease mechanism in TBI, CTE and AD, which can be blocked by *cis* mAb

Immunotherapy against tau is attracting attention because active or passive immunization against p-tau tangle epitopes reduces the initial formation of pathological tau ‘seeds’ and subsequent spreading of tau aggregates. Although extensive tangles are a neuropathological signature in CTE in sport and military, evidence of tau pathology is not readily detectable acutely after TBI in humans and mouse models using other commonly known antibodies/techniques. However, we found robust *cis* p-tau in human CTE brains. Surprisingly, after TBI in mice and stress in vitro, neurons acutely produce *cis* p-tau notable at axons, which disrupts axonal microtubules and axonal mitochondrial transport, spreads to other neurons, and leads to apoptosis [43]. This process, which we term ‘cistauosis’, appears long before other tau oligomers and fibrils but can be blocked by *cis* mAb and enhanced by *trans* mAb [43]. Treating TBI mice with *cis* mAb blocks early cistauosis, prevents the development and spread of tau aggregates such as oligomers and fibrils, and restores many TBI-related structural and functional changes. Thus, *cis*, but not *trans*, p-tau is a likely pathogenic form of tau in MCI and AD and the development of humanised antibodies directed against pathogenic *cis* p-tau could be useful for the early diagnosis and treatment of AD, TBI and CTE.

## Treatments to reduce pathogenic tau isoforms in AD, TBI and CTE

Currently there are no effective treatment options for patients with AD. Drug trials targeting reduction of the amyloid beta deposits have been shown to be efficacious in reducing amyloid beta deposits in the brain but there has been no convincing evidence that there is improvement in cognitive function [45]. The disappointing results from amyloid beta treatment studies has caused a shift in focus to the treatment of tau in AD. More recently, CSF biomarker studies have indicated that tau is superior to amyloid beta markers in following the progression of cognitive deficits in AD and other tauopathies [46, 47]. Furthermore, a newly developed PET technique to track changes in tau deposition in living patients with AD also shows that tau deposition more closely tracked dementia status and was a better indicator of cognitive deficits, thus indicating that treatments targeting tau could provide a superior alternative to amyloid beta therapies [45]. Several tau-targeting treatments have been trialled, or are currently being trialled. Most of these focus primarily on kinase inhibition or the inhibition of tau aggregation [48].

These strategies are likely to have a huge benefit for the treatment of AD and other tauopathies if proven successful but may be limited to treating only patients in the later stages of disease, especially those targeting tau aggregations. Since we find increases in *cis* p-tau early after TBI in both mouse and human brain, humanisation of our *cis* p-tau antibody for use as an early therapy could prove to be an effective intervention for patients early after TBI. Furthermore, it could potentially prevent the development of CTE following multiple head traumas. Finally, we have already shown that treatment with our *cis* p-tau antibody in mouse models not only prevents the development of neurofibrillary tangles and other tau pathologies, it also effectively removes *cis* p-tau aggregations and improved cognitive function. Therefore, further development and humanisation of the *cis* p-tau antibody also provides a potential new treatment for prevention and treatment of AD.

## Conclusion

The emergence of *cis* p-tau as a newly identified pathogenic isoform of tau presents a new pathway through which to develop new diagnostic tests to identify tau pathology following TBI, CTE and to identify early stage AD. Furthermore, the *cis* p-tau antibody targets a specific isoform of pathogenic tau which could ultimately be developed into a new treatment method for reducing tau pathology in AD, TBI and CTE and subsequently improving cognitive function in these patients.

## Abbreviations

AD: Alzheimer’s disease
ALS: amyotrophic lateral sclerosis
FTD: frontotemporal dementia
NFTs: neurofibrillary tangles
PSP: progressive supranuclear palsy
CBD: corticobasal dementia
CTE: chronic traumatic encephalopathy
TBI: traumatic brain injury
rmTBI: repeated mild traumatic brain injury
Aβ: Amyloid beta peptide 42 kDa
*cis* p-tau: *cis* isoform of phosphorylated tau protein
*trans*-p-tau: *trans* isoform of phosphorylated tau protein
Pin1: peptidyl, prolyl isomerase, Pin1
